# Skin cancer outcomes and risk factors in renal transplant recipients: Analysis of organ procurement and transplantation network data from 2000 to 2021

**DOI:** 10.3389/fonc.2022.1017498

**Published:** 2022-11-24

**Authors:** Xiaowei Hao, Wenhui Lai, Xinze Xia, Junnan Xu, Yangyang Wu, Chao Lv, Qingyang Meng, Kaikai Lv, Shuai Huang, Zhenjun Luo, Jun Dong, Qing Yuan

**Affiliations:** ^1^ Department of Urology, The Third Medical Center, Chinese PLA General Hospital, Beijing, China; ^2^ Department of Urology, No.971 Hospital of PLA Navy, Tsingtao, Shandong, China; ^3^ Department of Postgraduate, Hebei North University, Zhangjiakou, Hebei, China; ^4^ Department of Urology, Shanxi Medical University, Taiyuan, Shanxi, China; ^5^ Affiliated Hospital of Weifang Medical University, School of Clinical Medicine, Weifang Medical University, Weifang, China

**Keywords:** skin cancer, renal transplantation (RT), end stage renal disease (ESRD), UNOS/OPTN, risk factors

## Abstract

**Purpose:**

Posttransplant skin cancer is the most common malignancy after patients have undergone renal transplantation. Through comprehensive observation with a large sample size nationwide, understanding the risk factors and outcome of posttransplant skin cancer will help to develop appropriate patient surveillance and disease prevention strategies.

**Materials and methods:**

This retrospective population-based cohort study was based on Organ Procurement and Transplantation Network data released in March 2021. Characteristics and outcomes, including patient survival and graft survival of recipients, were compared. Risk factors for posttransplant skin cancer, cancer onset momentum, and mortality were determined.

**Results:**

A total of 199,564 renal transplant recipients were included. After renal transplantation, 7,334 (3.68%), 6,093 (3.05%), and 936 (0.47%) were diagnosed with squamous cell carcinoma, basal cell carcinoma, and melanoma, respectively. Skin cancer was the major cause of death (squamous cell carcinoma: 23.8%, basal cell carcinoma: 18%, and melanoma: 41.6%). Five-year survival rates ranked from best to worst were as follows: basal cell carcinoma (96.7 [95% confidence interval: 96.3–97.2]%), squamous cell carcinoma (94.1 [93.5–94.6]%), melanoma (89.7 [87.7–91.6]%), and cancer-free (87.4 [87.2–87.5]%) (*p* < 0.001 for all except melanoma vs. cancer-free, *p* = 0.534). Regarding graft survival, death-censored graft survival, posttransplant skin cancer, and melanoma were significantly better than the cancer-free group (*p* < 0.001). Independent risk factors for developing posttransplant skin cancer included older age, male sex, Caucasian race, pretransplant malignancy, polycystic kidney disease-induced end-stage renal disease (ESRD), retransplantation, private health insurance, T-cell depletion induction, and tacrolimus/mycophenolic acid use. Caucasian race and pretransplant malignancy were independent risk factors for posttransplant skin cancer onset momentum. Male sex, Caucasian race, pretransplant malignancy, hypertension- or diabetes-induced ESRD, retransplantation, diabetes history, deceased donor, cyclosporin, and mTOR inhibitor use were independent risk factors for posttransplant skin cancer mortality.

**Conclusion:**

Although posttransplant skin cancer is a major cause of recipient death, information regarding its impact on patient and graft survival is limited. Given the differences regarding risk factors for posttransplant skin cancer incidence, onset momentum, and mortality, personalized approaches to screening may be appropriate to address the complex issues encountered by kidney transplant recipients.

## Introduction

Renal transplantation (RT) is the most effective treatment for end-stage renal disease (ESRD). However, posttransplant malignancy is the leading cause of death and allograft loss, reducing the recipient’s lifespan and requiring heightened surveillance (1–3). Skin cancer, commonly categorized as non-melanoma skin cancer (NMSC) and melanoma according to tumor incidence, recurrence, metastasis, and aggressiveness (4, 5), represents a major threat to RT recipients. NMSC, primarily squamous cell carcinoma (SCC) and basal cell carcinoma (BCC), is the most frequent posttransplant malignancy, with a total incidence of 7.5%, which is up to 100-fold greater than that in the general population (6–11). Melanoma has a posttransplant incidence 1.5–2.5 times greater than that during dialysis (12–15) and is associated with high mortality and metastasis, comprising only 4% of cutaneous malignancies, but accounting for 80% of skin cancer deaths in the general population (16, 17).

Several studies have been conducted on posttransplant skin cancer (PTSC). Ponticelli et al. reviewed PTSC regarding its histopathological classification, characteristics, and treatment (18). In the general population, SCC is the second most common cancer in Caucasians, outnumbered by BCC at a 4:1 ratio (19). However, in transplant recipients, this ratio is reversed; the incidence of SCC is higher, followed by BCC (16, 17). This may reflect different pathogenic mechanisms underlying the development of these tumors in kidney transplant recipients. Ascha et al. highlighted the risk factors for melanoma and reported a greater risk of melanoma in American RT recipients than in the general population (20). Immunosuppressive therapy, skin color, and viral infection are widely recognized risk factors for skin cancer (17). Immunosuppressive agents are known to accelerate melanoma development in transplant recipients (21), whereas NSMC is closely related to sun exposure [especially ultraviolet (UV) radiation] (6, 22). Genetic factor as a fundamental cause of oncogenic activity should not be ignored. Laing et al. revealed that methylene tetrahydrofolate reductase (MTHFR) polymorphism plays a critical role in the elevated rate of SCC in renal transplant recipients (23, 24). Nevertheless, research in this field has mainly focused on the prevalence and risk factors for skin cancer after RT, but has not explored further aspects. Other studies were limited to one kind of skin cancer, NMSC or melanoma, and lack comprehensive comparison of different kinds of skin cancer.

Hence, we conducted a retrospective population-based cohort study using data from the Organ Procurement and Transplantation Network (OPTN) that includes three pathological types of PTSC. The study aimed to compare the outcomes of recipients and grafts and to identify risk factors for PTSC. We explore the impact of risk factors on PTSC development and outcomes. Our findings will improve our understanding of PTSC and could aid in the development of strategies to prevent or treat skin cancers in RT recipients.

## Methods

### Data source, study design, and participants

**Figure 1:**
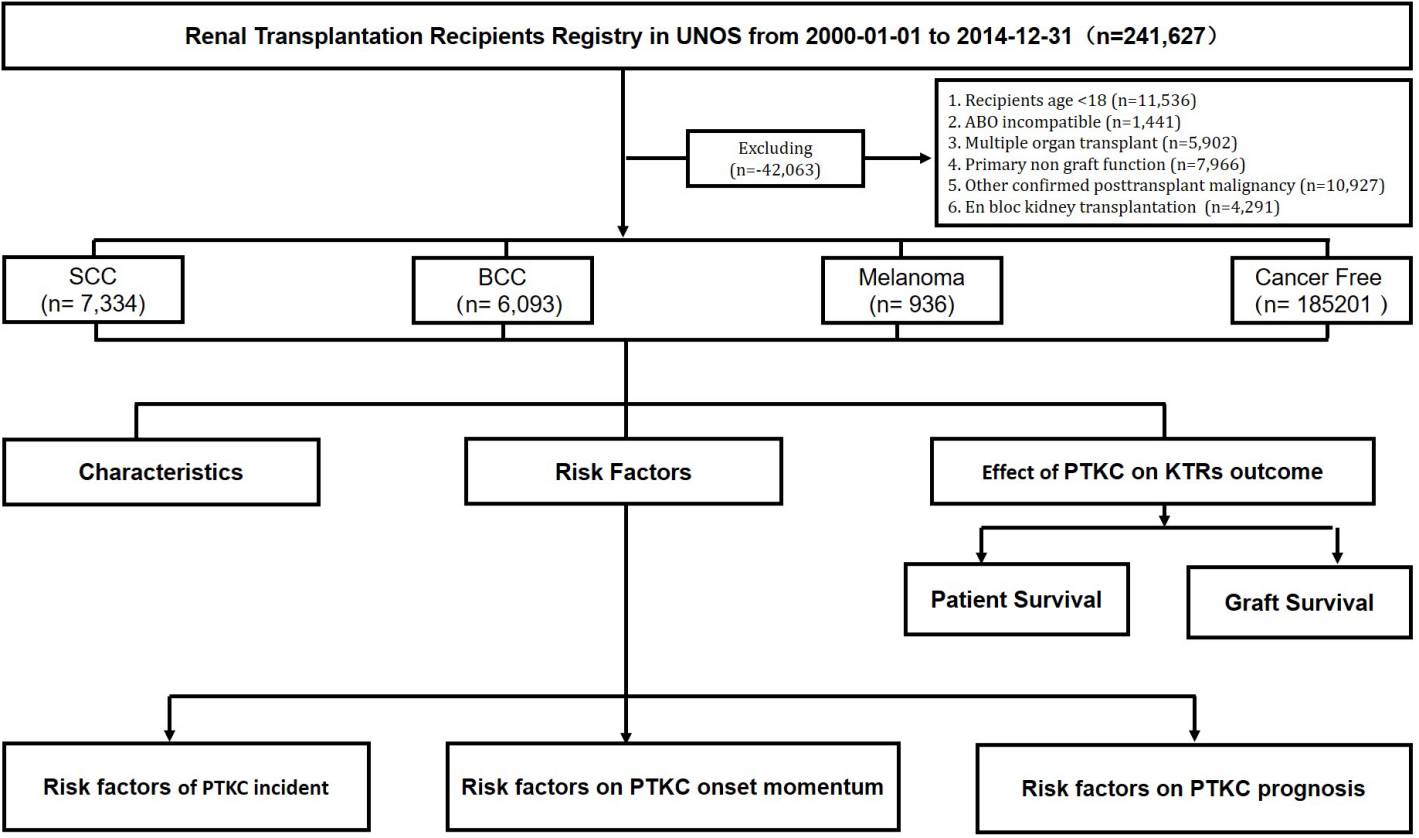
Flowchart of study cohort identification. SCC, squamous cell carcinoma; BCC, basal cell carcinoma. All kidney transplant recipients from 1 January 2000 to 31 December 2014 were followed from the date of transplantation to the date of outcome, censored for loss to follow-up, or end of the study period.

We analyzed data from the OPTN Standard Transplant Analysis and Research file released in March 2021. This is a retrospective population-based cohort study that included all adult kidney transplant recipients who underwent transplantation between 2000 and 2014 in the United States. Recipients who were <18 years old; who were ABO incompatible; who received multiorgan transplants, primary non-functional grafts, and other confirmed post-transplant malignancies; and who underwent en bloc kidney transplantation were excluded from the analysis (**Figure 1**). This paper is the responsibility of the authors alone and does not necessarily reflect the views or policies of the Department of Health and Human Services, nor does the mention of trade names, commercial, or organizations imply endorsement by the U.S. government.

### Exposure and outcomes classification and assessment

Time to outcome was defined as the date from transplantation until the date of the specified outcome (patient death or graft failure), censored for loss to follow-up, or the end of the study period. Outcomes included patient survival (PS), graft survival (GS), and death-censored GS (DCGS). The patients were divided into SCC, BCC, melanoma, and cancer-free (control) groups (**Figure 1**).

### Statistical analysis

Patient demographics and clinical characteristics were compared using the chi-square test for categorical variables and Student’s *t*-test for continuous variables; the distributions of the variables approximated normality, matched by the probability of skin cancer exposure based on a multivariable logistic regression model with odds ratios. The survival analysis is presented as Kaplan–Meier curves and compared using log-rank tests. The impact of potential skin cancer risk factors on cancer onset momentum was accounted for in the multivariable linear regression model. Cox proportional hazards models were fitted to estimate hazard ratios for skin cancer patient outcomes after adjusting for most potential confounders. Lasso regression was used to further estimate the optimal value for penalization coefficient lambda 22 confounders: (AGE+GENDER+ETHCAT_FACTOR+BMI_CALC_FACTOR+MALIG_HISTORY_FACTOR+DIAG_KI_FACTOR+ABO_FACTOR+DIALYSIS_DURATION+NUM_PREV_TX_FACTOR+DIAB_FACTOR+PRI_PAYMENT_TRR_KI_FACTOR+AGE_DON+GENDER_DON+ETHCAT_DON_FACTOR+BMI_DON_CALC_FACTOR+DON_TY_FACTOR+INDUCTION_THERAPY_AT_DISCHARGE_IL2RA+INDUCTION_THERAPY_AT_DISCHARGE_TCELL+MAINTENANCE_THERAPY_AT_DISCHARGE_CSA+MAINTENANCE_THERAPY_AT_DISCHARGE_TAC+MAINTENANCE_THERAPY_AT_DISCHARGE_MPA+MAINTENANCE_THERAPY_AT_DISCHARGE_MTOR) was selected for the prediction model. All analyses were performed using RStudio software version 1.1.456 (R. RStudio, Inc., Boston, MA, USA). Statistical significance was set at *p* < 0.05, and all confidence intervals were set at a 95% threshold. Descriptive statistics were used to summarize and present the data.

## Results

From January 2000 to December 2014, 241,627 patients received renal transplant registration in the United Network for Organ Sharing (UNOS). A total of 199,564 RT recipients met the inclusion criteria, of whom 7,334 (3.68%), 6,093 (3.05%), and 936 (0.47%) were diagnosed with SCC, BCC, and melanoma after RT, respectively.

Compared with the cancer-free group, cancer groups were older (56.01–57.33 vs. 48.95 years), were more male (69.7%–74.1% vs. 59.8%), had a higher proportion of Caucasians (92.3%–95.5% vs. 51.6%), had lower creatinine at discharge (2.30 vs. 2.82 mg/dl), had more pretransplant malignancy history (12.7%–13.7% vs. 4.5%), and had more polycystic kidney disease (PKD)-induced ESRD (16.6%–19.9% vs. 8.6%), but fewer had diabetes (17.3%–24% vs. 24.5%)- or hypertension (13.6%–15.1% vs. 22.5%)-induced ESRD, had more blood type A (42.9%–45.5% vs. 37.1%) and type B (8%–10.3% vs. 13%) but less type O (41.6%–42.2% vs. 45.1%), had a shorter waiting time (1.42–1.52 vs. 1.93 years) and dialysis duration (1.91–2.09 vs. 3.13 years), had less diabetes history (24.3%–31.3% vs. 31.3%), had less viral infection history including hepatitis B virus (HBV; 1%–1.6% vs. 1.8%), cytomegalovirus (CMV; 4.7%–5.2% vs. 5.5%), and human immunodeficiency virus (HIV; 0%–0.2% vs. 0.4%), had a higher proportion of college degrees (53%–55.6% vs. 42.8%), and had more private insurance (49.4%–55.6% vs. 38.7%). Regarding donors, cancer groups exhibited lower Kidney Donor Profile Index scores (KDPI; 0.39–0.41 vs. 0.41), were older (41.72–41.93 vs. 39.25, years), were not male (48.8%–50.1% vs. 52.4%), had a significantly higher proportion of Caucasians (86.9%–90.2% vs. 68.6%), had less obesity (22.8%–23.6% vs. 24.2%), had less deceased donors (46.5%–50.9% vs. 61.2%), had more donor skin cancer history (0.5%–0.7% vs. 0.3%), and had less human leukocyte antigen (HLA) mismatch (66.8%–73.3% vs. 76.9%). With regard to immunosuppressant therapy at discharge, cancer groups used less T-cell-depleting antibodies (29.6%–31.4% vs. 28.3%) and mTOR inhibitors (5.2%–6.1% vs. 7.7%), but more tacrolimus (72.7%–74.9% vs. 71.8%) and mycophenolic acid (MPA; 84.3%–84.9% vs.81.9%) (**Table 1**).

**Table 1:** Characteristics of recipients and donors.

Characteristics		SCC (*n* = 7,334)	BCC (*n* = 6,093)	Melanoma(*n* = 936)	Cancer free (*n* = 185,201)	*p*-value
**Age, years, mean (SD)**		56.82 (10.61)	56.01 (11.06)	57.33 (11.42)	48.95 (13.74)	<0.001
**Sex, M, No. (%)**		5,112 (69.7)	4,281 (70.3)	694 (74.1)	110,812 (59.8)	<0.001
**Ethnicity, No. (%)**	**White**	6,768 (92.3)	5,816 (95.5)	892 (95.3)	95,513 (51.6)	<0.001
	**Black**	187 (2.5)	32 (0.5)	11 (1.2)	48,478 (26.2)	
	**Hispanic**	259 (3.5)	177 (2.9)	26 (2.8)	27,442 (14.8)	
**BMI >= 30 kg/m^2^, Y, No. (%)**		2,013 (27.4)	1,604 (26.3)	294 (31.4)	57,162 (30.9)	<0.001
**Creatinine at discharge, mg, mean (SD)**		2.31 (1.99)	2.30 (2.02)	2.30 (1.89)	2.82 (2.51)	<0.001
**Pretransplant malignancy, No. (%)**	**NSMC**	337 (4.6)	325 (5.3)	47 (5.0)	1,162 (0.6)	<0.001
	**Melanoma**	86 (1.2)	86 (1.4)	18 (1.9)	435 (0.2)	
	**NSMC and melonoma**	14 (0.2)	10 (0.2)	2 (0.2)	40 (0.0)	
	**Other malignancy**	494 (6.7)	457 (7.5)	61 (6.5)	6,648 (3.6)	
	**Non-cancer history**	6,403 (87.3)	5,215 (85.6)	808 (86.3)	176,916 (95.5)	
**Cause of ESRD, No. (%)**	**Glomerular diseases**	1,400 (19.1)	1,271 (20.9)	186 (19.9)	35,261 (19.0)	<0.001
	**Hypertension**	1,105 (15.1)	830 (13.6)	129 (13.8)	41,609 (22.5)	
	**Polycystic kidneys**	1,221 (16.6)	1,211 (19.9)	159 (17.0)	15,976 (8.6)	
	**Diatetes**	1,418 (19.3)	1,056 (17.3)	225 (24.0)	45,302 (24.5)	
	**Retransplantion/graft failure**	1,087 (14.8)	772 (12.7)	96 (10.3)	20,385 (11.0)	
**Blood type**	**A**	3,195 (43.6)	2,612 (42.9)	426 (45.5)	68,735 (37.1)	<0.001
	**B**	712 (9.7)	626 (10.3)	75 (8.0)	24,084 (13.0)	
	**AB**	348 (4.7)	282 (4.6)	46 (4.9)	8,944 (4.8)	
	**O**	3,079 (42.0)	2,573 (42.2)	389 (41.6)	83,438 (45.1)	
**Waiting time, years, mean (SD)**		1.52 (1.55)	1.48 (1.53)	1.42 (1.46)	1.93 (1.89)	<0.001
**Dialysis duration, years, mean (SD)**		2.09 (2.93)	1.91 (2.72)	2.05 (2.65)	3.13 (3.21)	<0.001
**Transfusion history, Y, No. (%)**		1,506 (20.5)	1,114 (18.3)	186 (19.9)	37,648 (20.3)	0.006
**Diabetes, Y, No. (%)**		1,985 (27.1)	1,481 (24.3)	293 (31.3)	57,978 (31.3)	<0.001
**HBV surface antigen, Positive, No. (%)**		99 (1.3)	96 (1.6)	9 (1.0)	3,412 (1.8)	0.001
**CMV IgM, Positive, No. (%)**		383 (5.2)	284 (4.7)	46 (4.9)	10,257 (5.5)	0.015
**HIV antibody serum status, Positive, No.(%)**		15 (0.2)	7 (0.1)	0 (0.0)	738 (0.4)	<0.001
**Education level, College degree, No. (%)**		3,887 (53.0)	3,385 (55.6)	518 (55.3)	79,332 (42.8)	<0.001
**Private health insurance ,No. (%)**		3,684 (50.2)	3,387 (55.6)	462 (49.4)	113,573 (38.7)	<0.001
**KDPI, mean (SD)**		0.41 (0.27)	0.40 (0.27)	0.39 (0.27)	0.41 (0.27)	0.002
**Donor age, years, mean (SD)**		41.93 (13.86)	41.72 (13.75)	41.34 (14.40)	39.25 (14.38)	<0.001
**Donor sex, M, No. (%)**		3,576 (48.8)	2,963 (48.6)	469 (50.1)	96,988 (52.4)	<0.001
**Donor ethnicity, No.(%)**	**White**	6,373 (86.9)	5,406 (88.7)	844 (90.2)	127,127 (68.6)	<0.001
	**Black**	339 (4.6)	233 (3.8)	34 (3.6)	24,472 (13.2)	<0.001
	**Hispanic**	485 (6.6)	351 (5.8)	46 (4.9)	25,686 (13.9)	<0.001
**Donor BMI >= 30 kg/m2, Y, No. (%)**		1,694 (23.1)	1,390 (22.8)	221 (23.6)	44,808 (24.2)	0.014
**Donor type, Deceased, No. (%)**		3,684 (50.2)	3,831 (46.5)	476 (50.9)	113,435 (61.2)	<0.001
**Donor skin cancer history, Y, No. (%)**		36 (0.5)	43 (0.7)	6 (0.6)	582 (0.3)	<0.001
**HLA mismatch >= 3, Y, No. (%)**		5,375 (73.3)	4,373 (71.8)	625 (66.8)	142,479 (76.9)	<0.001
**Immunosuppressant at discharge, No. (%)**	**IL-2**	3,858 (52.6)	3,147 (51.6)	473 (50.5)	94,851 (51.2)	0.112
	**T-cell depleting**	2,169 (29.6)	1,848 (30.3)	294 (31.4)	52,496 (28.3)	<0.001
	**Cyclosporin**	960 (13.1)	908 (14.9)	150 (16.0)	27,397 (14.8)	0.001
	**Tacrolimus**	5,495 (74.9)	4,428 (72.7)	684 (73.1)	132,901 (71.8)	<0.001
	**MPA**	6,181 (84.3)	5,173 (84.9)	794 (84.8)	151,704 (81.9)	<0.001
	**mTOR inhibitor**	384 (5.2)	374 (6.1)	50 (5.3)	14,318 (7.7)	<0.001

SCC, squamous cell carcinoma; BCC, basal cell carcinoma; PTSC, posttransplant skin cancer; SD, standard deviation; M, male; Y, yes; BMI, body mass index; ESRD, end-stage renal disease; HBV, hepatitis B virus; CMV, cytomegalovirus; HIV, human immunodeficiency virus; KDPI, kidney donor profile index; HLA, human leukocyte antigen; IL-2, IL-2 receptor antibody; MPA, mycophenolic acid; mTOR, mammalian target of rapamycin.

Melanoma patients were the oldest (57.33 [11.42] years), were mostly male (74.1%), were the most obese (31.4%), had the most pretransplant malignancy (13.7%) and skin cancer (6.9%) histories, had a history of diabetes (31.3%), had the lowest KDPI (0.39 [0.27]), had the lowest HLA mismatch (66.8%), and had the lowest acute rejection incidence rate (0.7%). Among NSMC, there were no significant differences between baseline SCC and BCC rates (**Table 1**).

The skin color of the patients was associated with the incidence of skin cancer. The proportion of Caucasian patients with skin cancer was 40% higher than that in cancer-free patients. Interestingly, in the donor cohort, the proportion of Caucasians in the skin cancer group was 20% higher than that in the cancer-free group (**Table 1**). To clarify the significant proportion difference in donor ethnicity, we analyzed the proportion of recipient ethnicity who received Caucasians’ donor kidney. Compared to the cancer-free group, there is an obviously higher proportion of Caucasians who received kidney from a Caucasian donor (95.8%–97.1% vs. 65.7%), while a lower proportion of Blacks received kidney from a Caucasian donor in the skin cancer group (0.6%–1.5% vs. 20.6%). Furthermore, in both living donors and deceased donors, Caucasian recipients in skin cancer groups are more likely to receive a Caucasian donor kidney than those in the cancer-free group (living donor, 51.4%–57.2% vs. 34.5%; deceased donor, 40.0%–45.3% vs. 31.1%) (**Supplementary Table 1**). Regarding proportion of cancer type in ethnic groups, the most common type of PTSC in Blacks is SCC (81.3%). Pretransplant malignancy was also a risk factor for skin cancer incidence after transplantation. The incidence rate of skin cancer history in the cancer group was 5% higher than that in the cancer-free group and nearly 10% higher in terms of total malignancy history (**Table 1**).

### Effect of *de novo* kidney cancer on kidney transplant outcome

At the most recently reported follow-up, compared with the cancer-free group, the PTSC group had the highest skin cancer mortality (23.8%, 18%, and 41.6%) and exhibited a significantly lower incidence of delayed graft function and acute rejection, a longer follow-up period, and a higher death rate. SCC had the slowest cancer onset speed (6.0 [3.5, 9.2] years). BCC showed the longest follow-up period (IQR, median, 10.7 [7.7, 14.0] years) and lowest death rate (25.3%), even lower than those of cancer-free patients. Melanoma presented the worst outcome, including the shortest follow-up period (9.4 [6.8, 12.9] years), the fastest speed of cancer onset (4.9 [2.8, 8.2] years), and the highest death rate (42.6%). Melanoma displayed the lowest rate of graft function, while cancer-free patients exhibited the highest rate of graft failure (**Table 2**).

**Table 2:** Recipient status at most follow-ups with cause of death and graft failure.

Incidents		SCC(*n* = 7,334)	BCC(*n* = 6,093)	Melanoma(*n* = 936)	Cancer free(*n* = 185,201)	*p*-value
**Delayed graft function, Y, No. (%)**		763 (10.4)	565 (9.3)	91 (9.7)	29,538 (16.0)	<0.001
**Acute rejection, Y, No. (%)**		95 (1.3)	78 (1.3)	7 (0.7)	25,040 (13.5)	<0.001
**Follow-up period, years, median (IQR)**		9.9 (7.0, 13.5)	10.7 (7.7, 14.0)	9.4 (6.8, 12.9)	7.0 (4.4, 10.5)	<0.001
**PTSC diagnosed duration, years, median (IQR)**		6.0 (3.5, 9.2)	5.8 (3.2, 9.2)	4.9 (2.8, 8.2)	–	–
**Patient status, No. (%)**	**Alive**	4,397 (60.0)	4,114 (67.5)	477 (51.0)	102,582 (55.4)	<0.001
	**Retransplant**	140 (1.9)	163 (2.7)	18 (1.9)	13,004 (7.0)	
	**Lost to follow-up**	349 (4.8)	276 (4.5)	42 (4.5)	27,555 (14.9)	
	**Dead**	2,448 (33.4)	1,540 (25.3)	399 (42.6)	42,060 (22.7)	
**Cause of death, No. (%)**	**Other/Unknown**	1,273 (52.0)	844 (54.8)	162 (40.6)	25,839 (61.4)	<0.001
	**Skin cancer**	583 (23.8)	277 (18.0)	166 (41.6)	936 (2.2)	
	**Cardiocerebrovascular**	354 (14.5)	273 (17.7)	35 (8.8)	9,038 (21.5)	
	**Infection**	220 (9.0)	136 (8.8)	32 (8.0)	5,887 (14.0)	
	**Graft failure**	18 (0.7)	10 (0.6)	4 (1.0)	360 (0.9)	
**Graft status, No. (%)**	**Fuctioning**	4,289 (58.5)	4,038 (66.3)	466 (49.8)	105,103 (56.8)	<0.001
	**Partial faiture**	2,255 (30.7)	1,419 (23.3)	359 (38.4)	37,613 (20.3)	
	**Failure**	790 (10.8)	636 (10.4)	111 (11.9)	42,485 (22.9)	
**Cause of graft failure, No. (%)**	**Other/Unknown**	323 (4.4)	271 (4.4)	43 (4.6)	23,614 (12.8)	<0.001
	**Rejection**	25 (0.3)	13 (0.2)	1 (0.1)	1,380 (0.7)	
	**Primry nonfunction**	37 (0.5)	29 (0.5)	4 (0.4)	2,284 (1.2)	
	**Infection**	2 (0.0)	1 (0.0)	0 (0.0)	206 (0.1)	
	**Graft Thrombosis**	6,947 (94.7)	5,779 (94.8)	888 (94.9)	157,717 (85.2)	

SCC, squamous cell carcinoma; BCC, basal cell carcinoma; PTSC, posttransplant skin cancer; Y, yes; IQR, interquartile range; PTSC diagnosed duration: interval between transplantation and skin cancer diagnosis.

**Figure 2:**
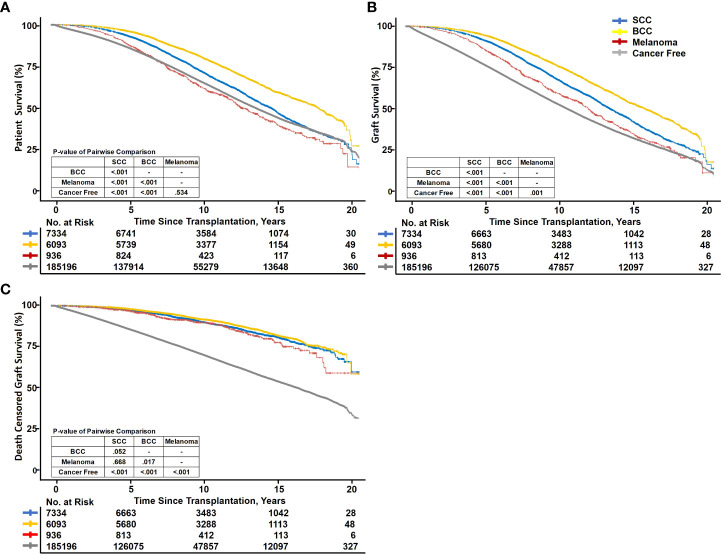
Kaplan–Meier survival curve fit of recipient and graft survival. SCC, squamous cell carcinoma; BCC, basal cell carcinoma. Kaplan–Meier curves showing patient survival **(A)**, graft survival **(B)**, and death-censored graft survival **(C)** between PTSC and cancer-free kidney transplant recipients.

Kaplan–Meier curves depict that BCC and SCC had the best outcomes for both patients and grafts of the four groups, whereas cancer-free patients had the worst outcomes (**Figure 2**). The PS between SCC, BCC, melanoma, and cancer-free patients showed statistically significant differences. *p*-values of pairwise comparisons between the four groups also confirmed significant differences in crude survival (*p* < 0.001 for each pair), except cancer-free and melanoma (*p* = 0.534). The 5-year survival rates of the four groups ranked from best to worst were as follows: BCC (96.7 [95% confidence interval: 96.3–97.2]%), SCC (94.1 [93.5–94.6]%), melanoma (89.7 [87.7–91.6]%), and cancer-free (87.4 [87.2–87.5]%) (**Figure 2A**). Regarding GS, BCC (95.6 [95.1–96.2]%), SCC (92.8 [92.2–93.4]%), melanoma (88.4 [86.3–90.4]%), and cancer-free (78.9 [78.7–79.1]%) also displayed significant differences (*p* < 0.001 for each pairwise comparison). BCC and SCC still ranked as the first two best GSs, but melanoma exceeded the cancer-free group (**Figure 2B**). DCGS ranking of the four groups was parallel to GS; however, the *p*-values for pairwise comparisons between each cancer group increased. The differences between the three cancer groups were smaller (*p* = 0.052, *p* = 0.668, and *p* = 0.017), whereas the differences between the cancer groups and cancer-free groups were greater (*p* < 0.001) (**Figure 2C**).

**Figure 3:**
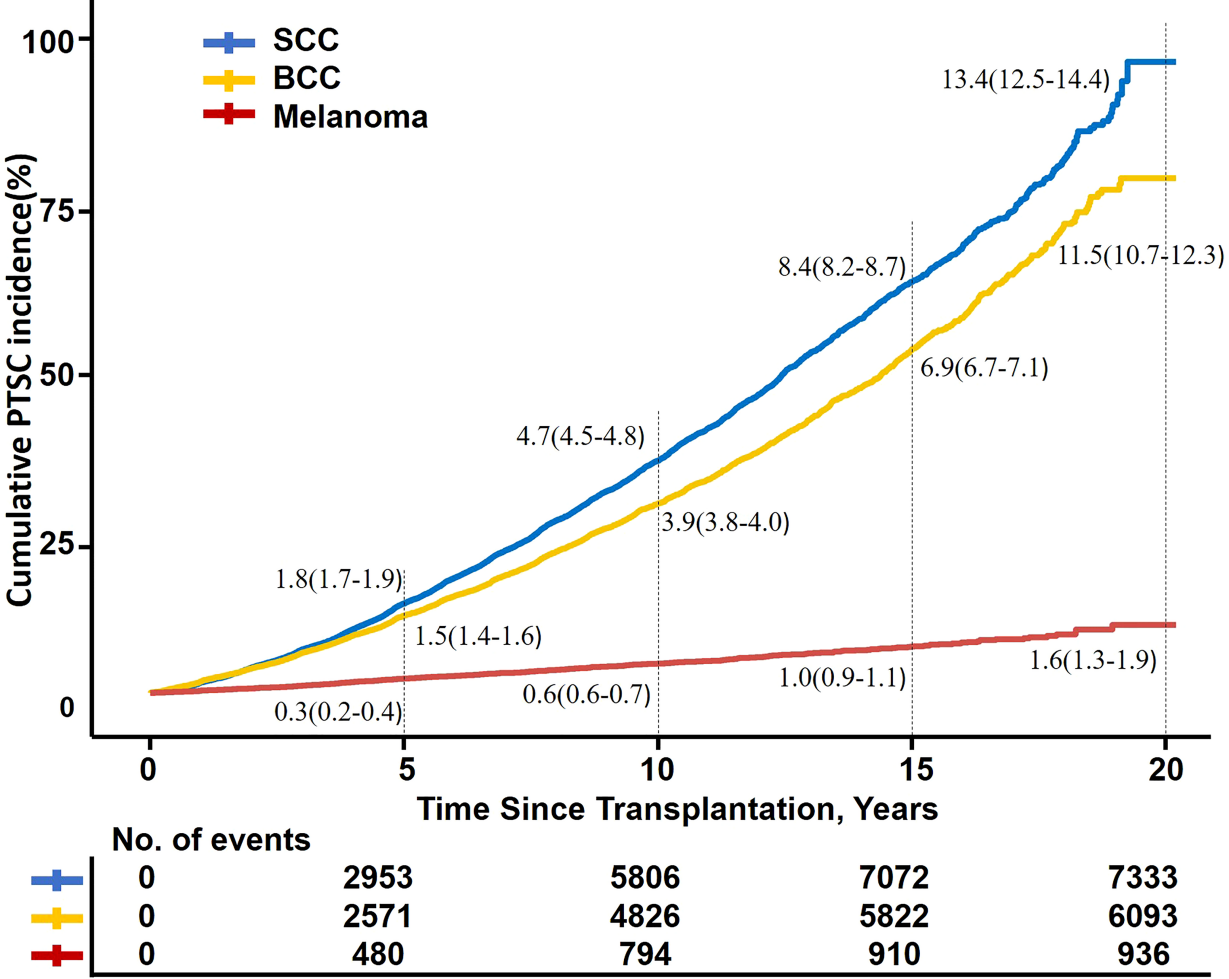
Kaplan–Meier fit of PTSC incidence by time after transplantation. SCC, squamous cell carcinoma; BCC, basal cell carcinoma; PTSC, posttransplant skin cancer.

Among the three types of skin cancer, the cumulative incidence of SCC and BCC was higher and the incidence rate increased with follow-up duration, while melanoma increased gradually with a lower incidence after RT (5- and 10-year cumulative incidence:1.8%, 4.7%; 1.5%, 4.9%; and 0.3%, 0.6%, respectively) (**Figure 3**).

### Risk factors of PTSC incidence

To identify unmodifiable factors at the time of transplantation that conferred a greater risk for subsequent malignancy within the transplant population, initial unadjusted analyses suggested that most variables were associated with a significantly increased risk of PTSC. After allowing for the effects of adjusted multivariate analysis, logistic regression model suggested that recipient characteristics such as older age (aOR: 1.05, 1.05, and 1.05, respectively), male (adjusted odds ratio: 1.56, 1.58, and 1.75, respectively), Caucasian (aOR: 9.95, 38.78, and 2.04, respectively), pretransplant malignancy (aOR: 1.63, 1.94, and 1.66, respectively), PKD-induced ESRD (aOR: 1.22, 1.30, and 1.21, respectively), retransplantation (aOR: 1.64, 1,29, and 1.13, respectively), private health insurance (aOR: 1.30, 1.48, and 1.33, respectively), Caucasian donor (aOR: 1.16, 1.30, and 1.61, respectively), T-cell depletion (aOR: 1.15, 1.20, and 1.08, respectively), tacrolimus (aOR: 1.21, 1.05, and 1.27, respectively), and MPA (aOR: 1.05, 1.22, and 1.10, respectively) were the independent risk factors for PTSC development. Obesity (aOR: 0.87, 0.83, NA, respectively), group B blood (aOR: 0.93, 1.00, 0.81, respectively), hypertension (aOR: 0.75, 0.70, 0.75, respectively), diabetes (aOR, 0.75, 0.72, 0.90, respectively)-induced ESRD, diabetes history (aOR: 0.85, 0.80, 0.81, respectively), deceased donor (aOR: 0.77, 0.76, 0.89, respectively), and mTOR inhibitor (aOR: 0.74, 0.87, 0.77, respectively) could decrease the risk of PTSC incidence. Moreover, other cofounders like IL-2 and longer dialysis duration exhibited differences that were not significant and would therefore not be discussed further (**Table 3**).

**Table 3:** Multivariable analysis of risk factors of PTSC incidence, onset, and outcome.

Risk factors		Logistic model(Odds ratio)			Linear model(Estemate)			COX model(Hazard ratio)		
		SCC	BCC	Melanoma	SCC	BCC	Melanoma	SCC	BCC	Melanoma
**Age**		**1.05**	**1.05**	**1.05**	**0.01**	**0.01**	**0.00**	**1.04**	**1.04**	**1.04**
**Sex, M**		**1.56**	**1.58**	**1.75**	**0.09**	**0.08**	**0.01**	**1.11**	**1.10**	**1.10**
**Ethnicity**	**Black**	**-**	**-**	**-**	**-**	**-**	**-**	**-**	**-**	**-**
	**White**	**9.95**	**38.78**	**2.04**	**0.28**	**0.25**	**0.04**	**1.16**	**1.16**	**1.18**
	**Hispanic**	**1.95**	**6.79**	**0.39**	**0.02**	**0.02**	**0.00**	**0.77**	**0.76**	**0.76**
**BMI >=30 kg/m^2^, Y**		**0.87**	**0.83**		**-0.04**	**-0.05**	**0.00**	**1.02**	**1.03**	**1.02**
**Malignancy history before TX**		**1.63**	**1.94**	**1.66**	**0.13**	**0.20**	**0.02**	**1.11**	**1.09**	**1.02**
**Cause of ESRD**	**Glomerular diseases**	**-**	**-**	**-**	**-**	**-**	**-**	**-**	**-**	**-**
	**Hypertension**	**0.75**	**0.70**	**0.75**	**-0.07**	**-0.08**	**-0.01**	**1.25**	**1.28**	**1.24**
	**Polycystic kidneys**	**1.22**	**1.30**	**1.21**	**0.11**	**0.02**	**0.02**	**0.78**	**0.76**	**0.77**
	**Diatetes**	**0.75**	**0.72**	**0.90**	**-0.08**	**-0.07**	**0.00**	**1.40**	**1.41**	**1.38**
	**Retransplant/graft failure**	**1.64**	**1.29**	**1.13**	**0.05**	**0.01**	**0.01**	**1.11**	**1.12**	**1.14**
**Blood type**	**AB**	**-**	**-**	**-**	**-**	**-**	**-**	**-**	**-**	**-**
	**A**	**×**	**×**	**1.06**	**×**	**×**	**0.00**	**1.01**	**1.02**	**1.01**
	**B**	**0.93**	**1.00**	**0.81**	**-0.01**	**0.00**	**0.00**	**1.00**	**0.99**	**1.00**
	**O**	**1.00**	**1.03**	**×**	**×**	**×**	**×**	**×**	**×**	**×**
**Dialysis duration**		**0.97**	**0.96**	**0.98**	**-0.01**	**-0.01**	**0.00**	**1.04**	**1.04**	**1.04**
**Diabetes, Y**		**0.85**	**0.80**	**0.81**	**-0.07**	**-0.07**	**-0.01**	**1.63**	**1.65**	**1.64**
**Private health insurance**		**1.30**	**1.48**	**1.33**	**0.06**	**0.08**	**0.01**	**0.76**	**0.75**	**0.75**
**Donor age**		**1.18**	**1.00**	**1.00**	**×**	**0.00**	**×**	**1.01**	**1.01**	**1.01**
**Donor sex, M**		**0.99**	**×**	**×**	**×**	**0.00**	**×**	**0.98**	**0.98**	**0.98**
**Donor ethnicity**	**Black**	**-**	**-**	**-**	**-**	**-**	**-**	**-**	**-**	**-**
	**White**	**1.16**	**1.30**	**1.61**	**0.05**	**0.07**	**0.01**	**0.92**	**0.91**	**0.93**
	**Hispanic**	**×**	**1.03**	**×**	**0.01**	**0.02**	**×**	**0.89**	**0.88**	**0.88**
**Donor BMI >= 30 kg/m2, Y**		**×**	**1.02**	**1.03**	**-0.01**	**-0.01**	**0.00**	**1.00**	**×**	**×**
**Donor Type, Deceased**		**0.77**	**0.76**	**0.89**	**-0.09**	**-0.08**	**-0.01**	**1.38**	**1.38**	**1.37**
**Immunosuppression at discharge**	**IL-2**	**1.02**	**1.10**	**1.03**	**0.00**	**0.00**	**-0.01**	**×**	**×**	**×**
	**T-cell depleting**	**1.15**	**1.20**	**1.08**	**0.00**	**0.00**	**0.00**	**0.90**	**0.90**	**0.90**
	**Cyclosporin**	**×**	**×**	**1.27**	**0.04**	**0.05**	**0.01**	**1.22**	**1.22**	**1.22**
	**Tacrolimus**	**1.21**	**1.05**	**1.27**	**0.07**	**0.03**	**0.01**	**1.00**	**0.99**	**1.00**
	**MPA**	**1.05**	**1.22**	**1.10**	**0.02**	**0.04**	**0.00**	**×**	**×**	**×**
	**mTOR inhibitor**	**0.74**	**0.87**	**0.77**	**-0.01**	**0.02**	**0.00**	**1.20**	**1.20**	**1.21**

M, male; Y, yes; BMI, body mass index; ESRD, end-stage renal disease.

“-”: reference; “×”: filtered out; “ ”: NA.

### Risk factors for PTSC onset momentum

The linear regression model suggested that Caucasian (SCC: [0.28], BCC: [0.25]) and pretransplant malignancies (SCC: [0.13], BCC: [0.20]) were associated with a longer duration of NMSC diagnosis.

### Risk factors for PTSC mortality

Male (adjusted hazard ratio [aHR]: 1.15, 1.20, and 1.08, respectively), Caucasian (aHR: 1.16, 1.16, and 1.18, respectively), pretransplant malignancy (aHR: 1.11, 1.09, and 1.02, respectively), hypertension (aHR: 1.25, 1.28, and 1.24, respectively)- or diabetes (aHR: 1.40, 1.41, and 1.38, respectively)-induced ESRD, retransplantation (aHR: 1.11, 1.12, and 1.14, respectively), diabetes history (aHR: 1.63, 1.65, and 1.64, respectively), deceased donor (aHR: 1.38, 1.38, and 1.37, respectively), and cyclosporin (aHR: 1.22, 1.22, and 1.22, respectively) and mTOR inhibitor (aHR: 1.20, 1.20, and 1.21, respectively) use significantly increased the mortality of patients with PTSC, whereas Hispanic (aHR: 0.77, 1.76, and 1.76, respectively), PKD-induced ESRD (aHR: 0.78, 0.76, and 0.77, respectively), private insurance (aHR: 0.76, 0.75, and 0.75, respectively), Hispanic donor (aHR: 0.89, 0.88, and 0.88, respectively), and T-cell depletion (aHR: 0.9, 0.9, and 0.9, respectively) were associated with decreased mortality of patients with PTSC (**Table 3**).

## Discussion

It is important to acknowledge the PTSC from three disease dimensions of incidence, onset momentum, and prognosis (mortality). We found not only that the risk factors for three dimensions of PTSC were different, but also that the effects of some cofounders on PTSC incidence, onset momentum, and mortality were opposite. Patients with hypertension, diabetes-induced ESRD, deceased donor, or T-cell-depleting agents have a significantly lower incidence risk of PTSC but a poorer prognosis if diagnosed with PTSC. On the contrary, PKD-induced ESRD or mTOR inhibitor use will raise the patient’s PTSC risk but exhibit relatively better outcome. Regarding outcomes, PTSC, particularly BCC, exhibited better outcomes, while patients with melanoma showed comparable PS and better GS compared with cancer-free cancer RT recipients.

Skin carcinogenesis following RT is associated with several factors, including immunosuppressive therapy, UV exposure, light skin color, genetic and epigenetic factors, viral infection, and psychogenic factors (20, 25–27). Laing et al. depicted that SCC was featured by aberrant methylation of DNA, which appears related to polymorphisms of MTHFR (28). An international survey indicated that the incidence rates of skin cancer were observed to increase with age in all of the studied countries including the USA (29). Skin cancer is more common in light-skinned Caucasians than in persons with skin of color and is often associated with greater morbidity and mortality, but is related to a slower onset momentum because skin color would minimize the likelihood of early detection of these tumors (30). Patients with pretransplant malignancies are prone to PTSC, which was also reported in another study using UNOS data. Patients with a history of malignancies had a hazard ratio of 1.77 to develop first posttransplant malignancy and 1.23-fold risk of mortality (31). A population-based study assessed the incidence of NMSC in American men, and it was twice that in women. Clearly, sex-related hormonal differences may play key roles in UV-induced skin inflammation and cancer development (32, 33). Considering that graft kidneys are unlikely to affect the patient’s skin and Caucasian candidates are more likely to be paired with Caucasian donors in living donor donations, whether Caucasian donors are a risk factor need to be determined by further studies. Generally, lower socioeconomic status is associated with decreased sun protection practices (34–36), but recipients with private insurance in our study, which usually means greater socioeconomic status, displayed an elevated risk of PTSC, which may be explained by the fact that most private insurance owners are Caucasian. Meanwhile, Caucasians or private insurance owners have a relatively lower risk of mortality, resulting from timely therapy with a shorter delay between diagnosis and definitive surgery (37, 38). Previous studies showed that PKD is an independent risk factor for NMSC development after RT, but the mechanisms linking PKD and NMSC are unclear. T-cell-depleting agents increasing the risk of cancers, such as melanoma, have already been proven (39, 40). The maintenance regimen often includes a calcineurin inhibitor (tacrolimus or cyclosporin), an antiproliferative agent (MPA or azathioprine), and steroids, all which are known to increase the risk of cutaneous malignancies. Our analysis corroborates results from current literature (41). Ascha et al. reported that sirolimus was a risk factor for melanoma after RT; however, another prospective clinical study suggested that mTOR inhibitors could have a preventive effect on PTSC genesis (42). We found that patients who used mTOR inhibitors at discharge, an immunosuppressant well known for its antioncogenic effects (43, 44), had a significantly decreased risk of PTSC. Interestingly, obesity is associated with a decreased risk of developing non-melanoma skin cancers. On one hand, obese individuals may likely spend less time outdoors with less chronic sun exposure (45). On the other hand, the “obesity paradox” has revealed that obesity may potentially attenuate the magnitude of inflammation (46). The protective effect of metformin, an antidiabetic drug associated with decreased cancer risk, on skin cancer in patients with diabetes has been proven in a cohort study in Taiwan (47, 48). Nevertheless, diabetes negatively impacts the recipients’ health and results in a worse prognosis. Several plausible hypotheses have been proposed for the observed association between ABO blood groups and skin cancer risk. Celi´c et al. demonstrated that the AB blood group was significantly associated with the occurrence of NMSCs (49), whereas Xie et al. revealed an association between the non-O blood group and a decreased risk of each type of skin cancer (50). One study found that patients with deceased donor had a significantly lower risk of PTSC incidence compared with those who did not. In our study, recipients of kidney from a deceased donor had a 1.38 greater hazard of mortality compared with living donor recipients. This is partially discordant with prior studies, with patients from deceased donors experiencing greater incidence of cancer and mortality (3, 51).

Skin cancer is the most commonly diagnosed cancer in the United States, in both RT recipients and the general population (4, 52, 53), threatens RT recipients’ lifespan and quality (11, 16, 22), and is regarded as a contraindication to transplantation (54). Partially congruent with previous studies, we observed higher morbidity but moderate skin cancer mortality in RT recipients. Compared with cancer-free patients after transplantation, posttransplant NMSC patients exhibited better survival, even melanoma, which usually results in early metastasis and is highly aggressive, also presented comparable survival in this study. This may be explained by the following reasons: (1) Tumor patients may have an inactive (immune escape) immune system to favor tumor progression but a better immune tolerance, which would be beneficial in terms of reducing transplant kidney rejection (55). It could also explain the fact that PTSC patients show lower proportion of HLA mismatch, lower creatinine at discharge, lower rate of delay graft function, and acute rejection. (2) RT patients are more proactive and regular in the early stages after RT, resulting in early treatment. Considering that skin cancer rarely impacts the kidney or allograft, and recipients with PTSC were more likely to undergo closer medical follow-up and thorough surveillance strategies, these recipients exhibited better GS features than cancer-free patients. (3) Given the time of skin cancer diagnosis in our study was more than 5 years after transplantation, most PTSC patients experience immunosuppression, and reducing it would not significantly impair their immune balance. However, skin cancer, especially melanoma, remains a common cause of death in patients with PTSC. Several studies found that melanoma and NMSC after transplantation are common causes of substantial mortality (26, 56). Combined with evidence from our study, the mean time to developing PTSC after RT was approximately 10 years and PTSC incidence increased gradually. The epidemiological features and tendency of PTSC provide important perspectives on prevention and evaluation of PTSC over the long-term and allow clinicians to take subsequent active steps to achieve the expected benefit during the early follow-up period.

Notably, this study may provide a basis for PTSC screening and treatment, especially for risk stratification and the development of individualized strategies. Considering that PTSC does not obviously impact PS and GS, which was demonstrated in our study, routine screening for PTSC may not be necessary for all recipients, but individualized screening among high-risk individuals particularly in the early follow-up period, especially cancer history, may be a more suitable and cost-effective approach.

Our research is a comprehensive observation that enrolled a large sample size spanning 15 years of registry and 7–10 years of follow-up period nationwide. However, several limitations exist in this study. First, despite the relatively large sample size, we lack information regarding cancer risk after transplant failure and are limited to avoiding loss to follow-up, which might result in underestimation of PTSC mortality in RT recipients. Secondly, information regarding sun exposure, such as occupational or recreational pastimes and sun protective habits, was unavailable. Thirdly, granular patient information of the data, particularly as it relates to immunosuppressive scheme during follow-up, and skin cancer details such as grade, stage, and therapies are insufficiently detailed in the UNOS registry. Finally, we could not assess certain risk factors for melanoma because they were not captured in the UNOS database.

Older age, male, Caucasian recipients, having pretransplant malignancy, PKD-induced ESRD, retransplantation, private health insurance, use of T-cell depletion, tacrolimus, and MPA are risk factors of PTSC incidence. Obesity, B blood group, hypertension- or diabetes-induced ESRD, diabetes history, deceased donor, and mTOR inhibitor use decreased the risk of PTSC incidence. Despite PTSC being a major cause of recipient death, its impact on both PS and GS remains limited. Given the differences in individual risks for PTSC and overall prognosis, a personalized approach to screening may be an appropriate strategy to address the complex issues encountered by RT recipients.

## Data availability statement

Publicly available datasets were analyzed in this study. This data can be found here: https://optn.transplant.hrsa.gov/data/request-data/.

## Ethics statement

Ethical review and approval was not required for the study on human participants in accordance with the local legislation and institutional requirements. Written informed consent for participation was not required for this study in accordance with the national legislation and the institutional requirements.

## Author contributions

XH, WL, JD, and QY designed the study. XH and WL conducted the statistical analysis and wrote the manuscript. All authors contributed to the critical revision of the manuscript for intellectual content and revised the manuscript. All authors read and approved the final manuscript.

## Acknowledgments

We gratefully acknowledge research support of the OPTN/UNOS data and Dr. Nahel Elias of the Center for Transplantation Sciences and Division of Transplant Surgery, Department of Surgery, Massachusetts General Hospital, Boston, MA, USA, for providing guidance in data analysis.

## Conflict of interest

The authors declare that the research was conducted in the absence of any commercial or financial relationships that could be construed as a potential conflict of interest.

## Publisher’s note

All claims expressed in this article are solely those of the authors and do not necessarily represent those of their affiliated organizations, or those of the publisher, the editors and the reviewers. Any product that may be evaluated in this article, or claim that may be made by its manufacturer, is not guaranteed or endorsed by the publisher.

## Supplementary material

Supplementary Table 1 Recipient ethnicity and donor type in patient received caucasian donor kidney.

## Abbreviations

BCC, basal cell carcinoma; BMI, body mass index; DCGS, death-censored graft survival; ESRD, end-stage renal disease; OPTN, Organ Procurement and Transplantation Network; PKD, polycystic kidney disease; PTSC, post-transplant skin cancer; RT, renal transplantation; SCC, squamous cell carcinoma; UNOS, United Network for Organ Sharing.
